# CIRBP Ameliorates Neuronal Amyloid Toxicity via Antioxidative and Antiapoptotic Pathways in Primary Cortical Neurons

**DOI:** 10.1155/2020/2786139

**Published:** 2020-02-27

**Authors:** Fang Su, Shanshan Yang, Hongcai Wang, Zhenkui Qiao, Zhengyi Qu, Hong Zhao

**Affiliations:** ^1^Department of Neurology, The Fourth Affiliated Hospital of Harbin Medical University, Harbin, Heilongjiang, China; ^2^Department of Neurology, The First Affiliated Hospital of Harbin Medical University, Harbin, Heilongjiang, China

## Abstract

It is generally accepted that the amyloid *β* (A*β*) peptide toxicity contributes to neuronal loss and is involved in the initiation and progression of Alzheimer's disease (AD). Cold-inducible RNA-binding protein (CIRBP) is reported to be a general stress-response protein, which is induced by different stress conditions. Previous reports have shown the neuroprotective effects of CIRBP through the suppression of apoptosis *via* the Akt and ERK pathways. The objective of this study is to examine the effect of CIRBP against A*β*-induced toxicity in cultured rat primary cortical neurons and attempt to uncover its underlying mechanism. Here, MTT, LDH release, and TUNEL assays showed that CIRBP overexpression protected against both intracellular amyloid *β*- (iA*β*-) induced and A*β*_25-35_-induced cytotoxicity in rat primary cortical neurons. Electrophysiological changes responsible for iA*β*-induced neuronal toxicity, including an increase in neuronal resting membrane potentials and a decrease in K^+^ currents, were reversed by CIRBP overexpression. Western blot results further showed that A*β*_25-35_ treatment significantly increased the level of proapoptotic protein Bax, cleaved caspase-3, and cleaved caspase-9 and decreased the level of antiapoptotic factor Bcl-2, but were rescued by CIRBP overexpression. Furthermore, CIRBP overexpression prevented the elevation of ROS induced by A*β*_25-35_ treatment by decreasing the activities of oxidative biomarker and increasing the activities of key enzymes in antioxidant system. Taken together, our findings suggested that CIRBP exerted protective effects against neuronal amyloid toxicity via antioxidative and antiapoptotic pathways, which may provide a promising candidate for amyloid-based AD prevention or therapy.

## 1. Introduction

Alzheimer's disease (AD) is an irreversible age-related neurodegenerative disorder, mainly characterized by progressive memory loss and cognitive decline [1]. One of the typical histological hallmarks associated with AD brains is the extracellular plaque deposits of the amyloid *β* (A*β*) peptides, which is produced by the cleavage of the transmembrane amyloid precursor protein (APP) [2–4]. Studies showed that A*β* peptides rapidly self-aggregates into A*β* dimmers, fibrils, and amyloid plaques and induced neuronal apoptosis in the cultured neuron [5, 6]. On the other hand, recent reports also demonstrated the accumulation of intracellular A*β* (iA*β*), especially A*β*_1–42_, at the early stage of AD development, which occurs earlier than the appearance of A*β* plaques [7]. Direct evidence for iA*β* cytotoxicity is that microinjection of A*β*_1-42_ induces neuronal cell death in cultured human primary neurons [8]. This cytotoxicity effect of causing neuronal cell death is found to be more potent than extracellular A*β* [9]. Currently, the toxicity of A*β* peptides is thought to contribute to the neuronal loss in the cerebral cortex and hippocampus and is involved in the initiation and progression of AD [10]. Although the underlying mechanisms by which A*β* production leads to the cytotoxicity and neuronal loss remains elusive, searching for strategies that can ameliorate A*β* toxicity may be potentially beneficial to AD treatment.

Cold-inducible RNA-binding protein (CIRBP) is a stress-responsive gene, which belongs to a family of cold-shock proteins [11]. In addition to upregulation of CIRBP induced by hypothermia, the expression of CIRBP can also be regulated by other stress conditions, such as hypoxia, UV radiation, glucose deprivation, and osmotic pressure [11]. In response to stress, CIRBP generally modulates mRNA stability at the posttranscriptional level through its binding site on the 3′-untranslational region (UTR) of its targeted mRNAs [12, 13]. Recent studies have demonstrated that CIRBP exerts neuroprotective effects against H_2_O_2_-induced cell death through the Akt and ERK pathways in primary rat cortical neurons and neuro2a (N2a) cells [14–16]. The aim of the present study is to investigate how CIRBP reacts to the intracellular and extracellular A*β* treatment, and whether CIRBP can protect against A*β*-induced toxicity in cultured rat primary cortical neurons.

In the present study, we found that CIRBP protected against both intracellular amyloid *β*- (iA*β*-) induced and A*β*_25-35_-induced cytotoxicity in rat primary cortical neurons. These neuroprotective effects of CIRBP were mediated through antioxidative and antiapoptotic pathways.

## 2. Material and Methods

### 2.1. Culture of Primary Cortical Neurons

Newborn Sprague-Dawley (SD) rats were used in these experiments. After cervical dislocation and sterilization by immersion in 75% ethanol, the whole brains were taken out from the head. Cortical tissues then were dissected from the brains in Dulbecco's modified Eagle's medium (DMEM) (Invitrogen, Carlsbad, CA). The tissues were mechanically dissociated by gently chopping for about 15 times, and then were digested with 0.25% trypsin (Invitrogen) for 25 minutes at 37°C. After terminating the digestion with DMEM containing 10% fetal bovine serum, the mixture was gently triturated through the pipette to obtain single-cell suspension. The suspension then was filtered through nylon meshes and centrifuged at 500 g for 5 minutes. Single cells were resuspended in DMEM with 10% fetal bovine serum (FBS), 2 g/l HEPES, penicillin G (100 U/ml), and 100 *μ*g/ml streptomycin (Invitrogen, Carlsbad, CA) and plated at a density of 1 × 10^6^/ml on poly-L-lysine-coated plates or coverslips. To inhibit glia cell growth and increase the purity of neurons, 10 *μ*M cytosine arabinoside (Sigma) was added to the medium 24 h after plating. Cells were used for experiments 6 days after culture. All animal experiments were approved by the Animal Care and Use Committee of Harbin Medical University.

### 2.2. Adenovirus Infection

The rat CIRBP gene was PCR amplified according to the following primers and then was subcloned into the pAdTrack-CMV plasmid (a gift from Bert Vogelstein) [17] through KpnI and XhoI restriction sites. The forward primer is ATGGCATCAGATGAAGGCAA and the reverse primer is TTACTCGTTGTGTGTAGCATA. The human intracellular A*β*_1–42_ cDNA was synthesized directly, and then was also subcloned into pAdTrack through BglII and XhoI restriction sites. Adenovirus packaging and quality testing were performed in HEK293 cells. The neurons after 6 days in culture were infected by directly adding the adenovirus into the culture medium with an optimized multiplicity of infection (MOI) for 12~24 h. Cellular and biochemical experiments were performed 48 h after infection.

### 2.3. A*β*_25-35_ Treatments

A*β*_25-35_ and a control peptide A*β*_35-25_ are from Sigma. Before use, 2 mM A*β*_25-35_ stock solution was prepared by water and aged in a humidified chamber at 37°C for 5 days to obtain aggregates of A*β* peptides. The control peptide A*β*_35-25_ followed the same procedure. After 12 h infection of adenovirus, the cells were treated with 20 *μ*M A*β*_25-35_ or A*β*_35-25_ by directly adding to the medium.

### 2.4. ELISA Assay

ELISA assay was used to determine the concentrations of A*β*_1-42_ in the cultured rat cortical neurons after the infection of recombinant adenoviruses using the ELISA kit (R & D Systems) according to the manufacturer's instruction. The microplate reader (Bio-Rad) was used to evaluate the intensity of each well at 480 nm.

### 2.5. Cell Cytotoxicity Analysis

In this study, the cytotoxicity of the cells after A*β* treatment was assessed by MTT assay and lactate dehydrogenase (LDH) release assay. For MTT assay, cells were seeded in 96-well plates. After treatment, media of the culture neurons were carefully removed by aspiration. After gently washing with PBS, 100 *μ*l cell culture medium containing 0.5 mg/ml MTT was added to each well and incubated at 37°C for 4 h. Then, the medium in the wells was discarded and 150 *μ*l dimethyl sulfoxide was added to each well. The absorbance was measured at 570 nm using a microplate reader (Bio-Rad). LDH release assay was measure using a CytoTox 96® Non-Radioactive Cytotoxicity Assay kit (Promega). This experiment was performed according to the manufacturer's instructions.

### 2.6. Electrophysiology

The cortical neurons were bathed in an extracellular solution containing (in mM) 140 NaCl, 2.5 KCl, 1.2 MgCl_2_, 2 CaCl_2_, 1.2 NaH_2_PO_4_, 10 HEPES, and 10 glucose, pH 7.35 adjusted with NaOH. 1 *μ*M TTX and 100 *μ*M CdCl_2_ were also included in the bath solution to block voltage-gated Na^+^ and Ca^2+^ channels. The patch pipette solution contained (in mM) 115 K-gluconate, 5 KCl, 5 Na_2_-ATP, 2 MgCl_2_, 1 CaCl_2_, 10 EGTA, and 10 HEPES, pH 7.2 adjusted with KOH. Pipettes with a resistance of 3–5 M were used in this experiment. The neurons were held at -70 mV, and then depolarized in a whole-cell patch clamp configuration by 1000 ms from -60 to 80 mV with 10 mV steps using an Axon 200B amplifier at room temperature.

### 2.7. Western Blot Analysis

After washing with PBS for 2 times, the cultured cells were treated with cell lysis buffer to extract the total proteins. A total of 20 *μ*g of protein samples was separated on a 10% SDS-PAGE, and then transferred to a polyvinylidene fluoride (PVDF, Millipore) membrane (Millipore, Bedford, MA, USA). After blocking with 5% nonfat milk in Tris-buffered saline Tween-20 (TBST) for 1 h at room temperature, the PVDF membranes were incubated with the primary antibodies at 4°C overnight. After washing, the membranes were further incubated with horseradish peroxidase- (HRP-) conjugated secondary antibodies for 1 h at room temperature. *β*-Actin was used as a loading control. The intensities of the lanes were quantified using ImageJ software.

### 2.8. Cell Apoptosis Analysis

Terminal deoxynucleotidyl transferase-mediated dUTP nick end-labeling (TUNEL) assay (Roche) was used to evaluate the rate of cell apoptosis according to the manufacturer's instructions. Briefly, cells were fixed in 4% parafomaldehyde solution for 30 minutes at room temperature and permeabilized in 0.1% Triton X-100 for 5 min. TUNEL reagents then were added and incubated for 1 h at 37°C. After washing with PBS, the cells were mounted with DAPI in a mounting solution.

### 2.9. Caspase Activity Measurement

The activities of caspase-9 and caspase-3 were measured by using the fluorometric assay kit according to the manufacturer's instructions (Cell Signaling Technology). The protein samples were incubated with the reaction buffer and initiated by the DEVD-AMC substrate in a 96-well plate. The fluorescence intensity was measured using a fluorescence reader with excitation at 380 nm and emission at 460 nm.

### 2.10. Intracellular ROS Measurement

The measurement of intracellular ROS level was performed using a 2,7-dichlorofluorescein diacetate (DCF-DA) detection kit (Abcam) according to the manufacturer's instruction. Briefly, after washing twice with PBS buffer, adherent neurons were digested with 0.25% trypsin. Then, the cells were resuspended and incubated with 10 *μ*M DCF-DA at 37°C for 30 min. After staining, the DCF fluorescence was detected using a fluorescence spectroscopy with excitation/emission at 495 nm/529 nm (BD Biosciences).

### 2.11. ELISA

After different treatments, cortical neurons were homogenized in lysis buffer to obtain the protein extracts. The activities of superoxide dismutase (SOD), catalase (CAT), glutathione peroxidase (GPx), 4-hydroxy-2-nonenal (4-HNE), and malondialdehyde (MDA) were measured using ELISA kits (R&D Systems) according to the manufacturer's instructions.

### 2.12. Statistical Analysis

Results were expressed as mean ± SE. Statistical analysis was performed using Student's *t*-test or the Mann–Whitney rank sum test. A value of *p* < 0.05 was considered significant.

## 3. Results

### 3.1. CIRBP Overexpression Reduced A*β*-Induced Neurotoxicity in Rat Primary Cortical Neurons

**Figure 1:**
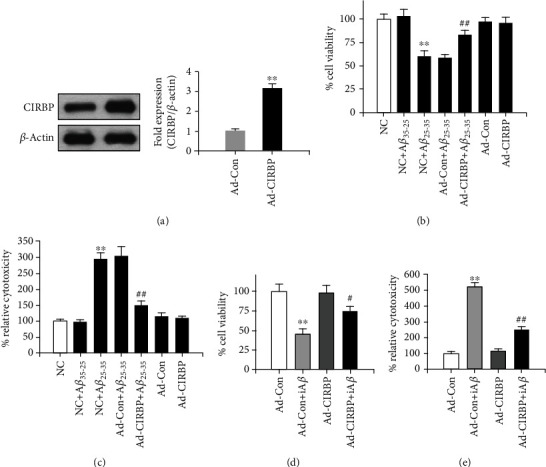
CIRBP protected against A*β*-induced neurotoxicity in rat primary cortical neurons. (a) Western blot result shows CIRBP overexpression in cultured cortical neurons. ^∗∗^*p* < 0.01*vs*. Ad-Con. (b) Cell viability was measured by MTT assay after treatment with 20 *μ*M A*β*_25-35_. (c) Cell cytotoxicity was tested by LDH release assay after treatment with 20 *μ*M A*β*_25-35_. ^∗∗^*p* < 0.01*vs*. NC, ^##^*p* < 0.01*vs*. Ad-Con+A*β*_25-35_. (d) Cell viability was measured by MTT assay after iA*β*_1-42_ adenovirus infection. (e) Cell cytotoxicity was tested by LDH release assay after iA*β*_1-42_ adenovirus infection. ^∗∗^*p* < 0.01*vs*. Ad-Con, ^#^*p* < 0.05, ^##^*p* < 0.01*vs*. Ad-Con+iA*β*.

To examine whether CIRBP could reduce A*β*-induced neurotoxicity, rat primary cortical neurons cultured for 6 days were infected with an adenovirus-carrying CIRBP gene (Ad-CIRBP) at a MOI of 2 for 24 h, and then exposed to 20 *μ*M A*β*_25-35_ or a control peptide A*β*_35-25_ for another 24 h. Cell viability was analyzed by MTT assay, and cell cytotoxicity was analyzed by LDH release assay. First, CIRBP expression was analyzed by western blot. In the Ad-CIRBP group, CIRBP expression was 3-fold higher than in the control group (Ad-Con) (Figure 1(a)). Although the biochemical organization of A*β*_25-35_ (monomeric or oligomeric) was not evaluated, MTT assay results showed that compared to the control peptide A*β*_35-25_, exposure to 20 *μ*M A*β*_25-35_ dramatically reduced cell viability to about 60%, which was prevented by CIRBP expression in the Ad-CIRBP group, but not in the Ad-Con group (Figure 1(b)). Notably, CIRBP overexpression itself has no effect on cell viability (Figure 1(b)). LDH release assay showed similar results (Figure 1(c)), suggesting that there is a neuroprotective effect of CIRBP against A*β*-induced toxicity.

Here, we also checked the effects of CIRBP on iA*β*-induced neurotoxicity. After infection with the adenovirus-carrying CIRBP gene for 24 h, rat primary cortical neurons were infected with another iA*β*_1-42_ adenovirus containing the human A*β*_1-42_ sequence without any signal peptide for another 24 h. As shown in a previous study [18], this construct mainly expresses A*β*_1-42_ in the cytosol and has higher toxicity than the construct coding human A*β*_1-42_ sequence with an addition signal peptide in human neurons. Our result in ELISA assay showed that the production of A*β*_1-42_ in the cultured rat cortical neurons after the infection of recombinant adenoviruses at the MOI of 0.1 was about 11.2 ng/ml (Supplemental Figure 1). Compared with the results above, iA*β*_1-42_ dramatically reduced cell viability to about 40% tested by MTT and increased cytotoxicity tested by LDH release assay in the Ad-Con+iA*β* group, showing more toxicity of iA*β*_1-42_ than A*β*_25-35_ (Supplemental Figure 2). However, this effects of iA*β*_1-42_ were significantly rescued in the Ad-CIRBP+iA*β* group (Figures 1(d) and 1(e)), although the form of intraneuronal A*β* (monomeric or oligomeric) was still unclear. These results indicated that CIRBP overexpression prevented against the iA*β*_1-42_-induced cytotoxicity.

### 3.2. CIRBP Overexpression Reversed iA*β*-Induced Electrophysiological Changes That Are Responsible for Neuronal Toxicity

**Figure 2:**
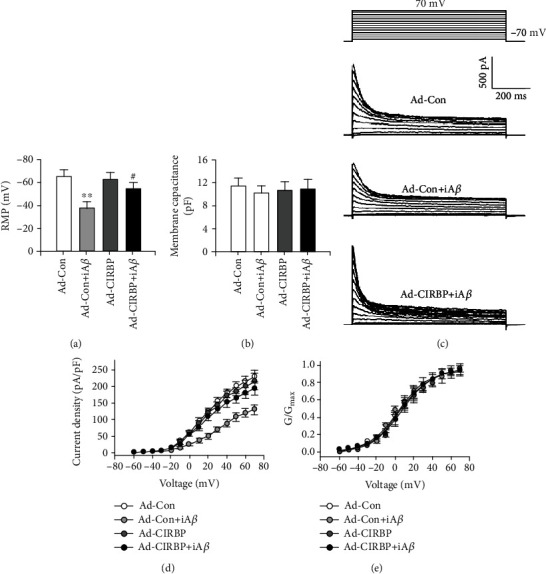
CIRBP reversed iA*β*-induced electrophysiological changes that are responsible for neuronal toxicity. (a, b) Resting membrane potentials (RMP) (a) and membrane capacitance (b) were recorded among different groups. ^∗∗^*p* < 0.01*vs*. Ad-Con, ^#^*p* < 0.05*vs*. Ad-Con+iA*β*. (c) Typical recordings of whole-cell K^+^ current in the Ad-Con, Ad-Con+iA*β*, and Ad-CRIPB+iA*β* groups by depolarizing the membrane from -70 mV to +70 mV from a holding potential at -70 mV with 10 mV increasing steps. (d) Current-voltage relationship of the peak K^+^ current density. ^∗∗^*p* < 0.01*vs*. Ad-Con. (e) Voltage-dependent activation curve of K^+^ current showing there was no difference among different groups.

The change of electrophysiological properties induced by A*β* treatment, such as an increase in neuronal resting membrane potentials and a decrease in K^+^ currents, is thought to be responsible for neuronal toxicity [18–22]. Here, to test whether CIRBP could reverse the iA*β*-induced electrophysiological changes, we recorded the resting membrane potentials and K currents in rat primary cortical neurons that were infected with iA*β*_1-42_ adenovirus with or without CIRBP overexpression. Our results showed that the resting membrane potentials dramatically increased after iA*β*_1-42_ treatment (−38.2 ± 4.5 mV) compared with the Ad-Con group (−64.8 ± 6.2 mV) (Figure 2(a)). Moreover, iA*β*_1-42_ also reduced the whole-cell K^+^ current density in the Ad-Con+iA*β* group (Figures 2(c) and 2(d)) but did not alter the voltage-dependent activation of K^+^ currents (Figure 2(e)). These results were consistent with previous studies [18, 20–22]. In this study, we found that CIRBP overexpression rescued the loss of resting membrane potentials (−55.4 ± 4.2 mV) and the decrease of K^+^ currents induced by iA*β*_1-42_ (Figures 2(a) and 2(d)). However, CIRBP overexpression itself had no effect on the resting membrane potentials (−63.1 ± 5.2 mV) and K^+^ current density (Figures 2(a) and 2(d)). The membrane capacitance did not change either in iA*β* treatment or CIRBP overexpression (Figure 2(b)). These results indicated that CIRBP ameliorated A*β*-induced neurotoxicity by rescuing the electrophysiological properties of the neurons.

### 3.3. CIRBP Overexpression Prevented A*β*-Induced Activation of Cell Apoptosis Pathway in Cortical Neurons

**Figure 3:**
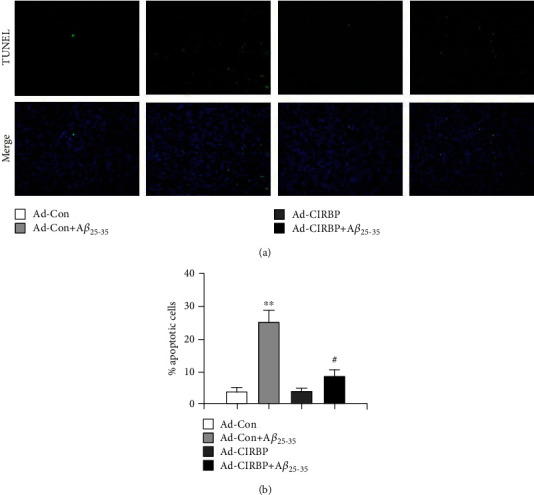
CIRBP prevented A*β*_25-35_-induced neuronal apoptosis. (a) Apoptotic cells were evaluated by TUNEL staining. Representative TUNEL staining images showing blue nuclear staining by DAPI and green TUNEL staining. (b) The percentage of apoptotic cells was calculated following the formula: Apoptosis index = apoptotic cells/(apoptotic cells + normal cells). ^∗∗^*p* < 0.01*vs*. Ad-Con, ^##^*p* < 0.01*vs*. Ad-Con+A*β*_25-35_.

To elucidate how CIRBP protected against A*β*-induced neurotoxicity in rat primary cortical neurons, we used TUNEL assay to measure the cell apoptosis in each group. Compared with the Ad-Con group, 20 *μ*M A*β*_25-35_ treatment significantly increased the percentage of cell apoptosis in the Ad-Con+A*β*_25-35_ group, while overexpression of CIRBP markedly attenuated cell apoptosis induced by A*β*_25-35_ (Figure 3). However, no significant difference was observed between the Ad-Con and Ad-CIRBP groups (Figure 3).

**Figure 4:**
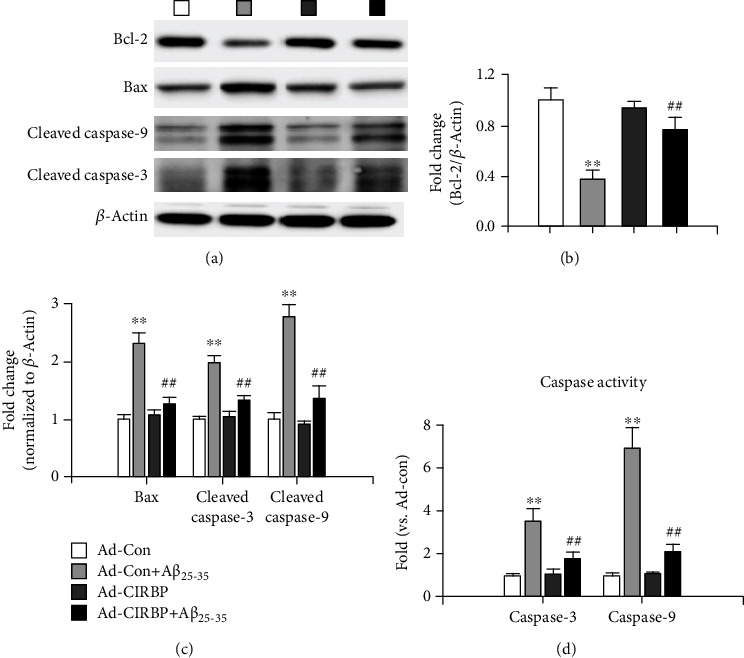
CIRBP inhibited A*β*_25-35_-induced neurotoxicity via the antiapoptotic pathway. (a) Representative imaging of western blot assay. (b) The expression level of Bcl-2 normalized to the expression level of *β*-actin. (c) The expression level of Bax, cleaved caspase-3, and cleaved caspase-9. (d) The caspase-3 and caspase-9 activities measured by a commercial fluorescent quantitative detection kit. ^∗∗^*p* < 0.01*vs*. Ad-Con, ^##^*p* < 0.01*vs*. Ad-Con+A*β*_25-35_.

Next, western blot was performed to evaluate the expression of some key apoptosis-related genes, such as antiapoptotic factor, B cell leukemia/lymphoma-2 (Bcl-2) and proapoptosis factors, Bcl-2 associated X protein (Bax), cleaved caspase-9, and cleaved caspase-3. As shown in Figures 4(a) and 4(b), the protein level of antiapoptotic factor, Bcl-2, was significantly decreased after A*β*_25-35_ treatment but was reversed by CIRBP overexpression. In contrast, the proapoptosis factors, Bax, cleaved caspase-9, and cleaved caspase-3, were all upregulated in the Ad-Con+A*β*_25-35_ group, which were inhibited by CIRBP overexpression (Figures 4(a) and 4(c)). We further measured the activities of caspase-9 and caspase-3 by using commercial fluorescent quantitative detection kit. Consistent with the western bolt result, 20 *μ*M A*β*_25-35_ treatment significantly increased the activities of caspase-9 and caspase-3, while CIRBP overexpression ameliorate this effect (Figure 4(d)). Thus, these results indicated that the underlying mechanism of CIRBP protective effect against neuronal amyloid toxicity may be mediated by the antiapoptotic pathway.

### 3.4. CIRBP Overexpression Inhibited A*β*-Induced Oxidative Stress in Cortical Neurons

**Figure 5:**
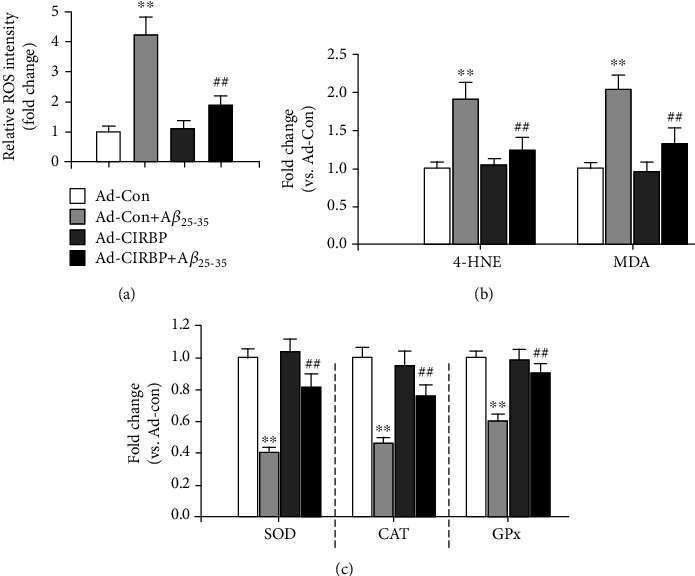
CIRBP inhibited A*β*_25-35_-induced neurotoxicity via the antioxidative pathway. (a) Intracellular ROS level was measured using a ROS-detecting fluorescence dye DCF-DA kit. (b) The activities of oxidative stress biomarkers, such as 4-HNE and MDA, measured by ELISA assay. (c) The activities of key enzymes in an antioxidant system, such as SOD, CAT, and GPx, were measured by ELISA. ^∗∗^*p* < 0.01*vs*. Ad-Con, ^##^*p* < 0.01*vs*. Ad-Con+A*β*_25-35_.

Oxidative stress, such as reactive oxygen species (ROS) generation, induced by A*β* is one of the major causes that lead to neuronal apoptosis [23]. Therefore, we further examined whether CIRBP could suppress A*β*-induced oxidative stress in cortical neurons. Intracellular ROS level in cortical neurons was assessed by using a ROS-detecting fluorescence dye DCF-DA kit. As expected, the level of ROS in cortical neurons treated with 20 *μ*M A*β*_25-35_ was significantly elevated to 4.3-fold compared with the Ad-Con group. However, CIRBP overexpression remarkably inhibited the ROS generation induced by A*β*_25-35_ (Figure 5(a)). Next, the activities of oxidative marker, such as 4-hydroxy-2-nonenal (4-HNE) and malondialdehyde (MDA) and key enzymes in the antioxidant system, such as superoxide dismutase (SOD), catalase (CAT), and glutathione peroxidase (GPx), were measured by ELISA. As shown in Figures 5(b) and 5(c), CIRBP overexpression significantly rescued A*β*_25-35_-induced upregulation of oxidative marker and A*β*_25-35_-induced downregulation of antioxidase activities of these three antioxidant enzymes, indicating that the antioxidative pathway mediated the protective effect of CIRBP against A*β*-induced neuronal apoptosis.

## 4. Discussion

AD is one of the neurodegenerative disorders in the elderly with extremely deficient clinical therapies and is associated with high morbidity and mortality [1]. AD is clinically characterized by progressive memory loss and cognitive decline [1]. One of the major pathological hallmarks in AD is the appearance of amyloid plaques that is enriched in A*β* [2–4]. Extensive studies suggest that the neurotoxicity of A*β* contributes to the neuronal loss and involves in the pathogenesis of neuronal dysfunction in AD [5, 24]. Although the underlying mechanisms are still largely unknown, accumulated evidences have showed that natural products or genetical manipulation that protect against A*β*-induced neuronal loss are beneficial to the treatment of AD [18, 25]. In the present study, we found that CIRBP, a stress-response protein, attenuated A*β*-induced cytotoxicity via antioxidative and antiapoptotic pathways in cultured rat primary cortical neurons, which may provide a promising candidate for amyloid-based AD prevention or therapy.

A*β*-mediated electrophysiological changes have been extensively studied in human primary neurons, rodent neurons, and different cell lines [18–22]. Dysfunction of neuronal excitability is responsible for the neuronal toxicity induced by A*β* [26]. Here, we found that iA*β* induced significant increase in resting membrane potential and decrease in K^+^ current density in the Ad-Con+iA*β* group, which is consistent with previous reports [18, 20–22]. Suppression of K^+^ current by A*β* was reported to trigger a large increase of Ca^2+^ influx by activating voltage-gated Ca^2+^ channels in the distal dendrites of rat hippocampal neurons [20]. The Ca^2+^ imbalance is suggested to initiate neuronal dysfunction and lead to cell death in rat hippocampus cells. In the present study, CIRBP overexpression rescued the resting membrane potential, probably by increasing the K^+^ current density, to maintain normal neuronal excitability. A previous report in the heart showed that CIRBP regulated cardiac repolarization by reducing the expression and function of transient outward K^+^ current [27]. Our present study identified a role of CIRBP in regulating another K^+^ channel, although the underlying mechanism still needs further investigation.

CIRBP, a cold-shock protein found in mammals, expressed in various cell types and is involved in multiple biological and cellular processes, such as cell survival, apoptosis, cell proliferation, circadian rhythm, immune response, reproduction, and cancer [11]. Upregulation of CIRBP at mild hypothermia is reported to suppress cell death and contribute to cell survival [16, 28, 29]. Li et al. first demonstrated that CIRBP upregulation in rat cortical neurons at low temperature inhibited H_2_O_2_-induced neuronal apoptosis [16]. Liu et al. also found that CIRBP protected against oxidative stress via the Akt and ERK signal transduction pathways in N2a cells [15]. Sakurai et al. observed that mild hypothermia-induced elevated CIRP levels inhibited tumor necrosis factor-alpha-induced apoptosis by caspase-8 activation and ERK phosphorylation [30]. Zhang et al. revealed a neuroprotective effect of CIRBP during mild hypothermia by inhibiting neuron apoptosis via the suppression of the mitochondria apoptosis pathway [14]. Consistent with these previous reports, we found that CIRBP overexpression significantly attenuated A*β*-induced neuronal apoptosis and exerted a neuroprotective effect in cultured rat cortical neurons. Mitochondrial signaling pathway plays an important role in promoting apoptotic cell death [31]. In our study, CIRBP overexpression rescued the decreased protein expression of antiapoptotic factor-Bcl-2 and also inhibited the increased protein expression of proapoptosis factor Bax, cleaved caspase-9, and cleaved caspase-3. All these results indicated that CIRBP suppressed A*β*-induced neuronal apoptosis, probably via mitochondrial signaling pathway.

A*β*-induced neuronal apoptosis is associated with the generation of ROS and plays an important role in the pathogenesis of AD [23]. Various compounds with antioxidant ability have been proposed to attenuate A*β*-induced oxidative stress in studies done *in vitro* and *in vivo* [32]. In this study, we showed that A*β*_25-35_ treatment dramatically elevated intracellular ROS levels in culture primary cortical neurons, which is consistent with previous studies [23, 32]. However, CIRBP overexpression largely prevented A*β*_25-35_-induced excessive ROS release, confirming the antioxidative activity of CIRBP. In addition, we evaluated the activities of primary antioxidant enzyme, such as SOD, CAT, and GPx. SOD can scavenge superoxide anions by catalyzing them to hydrogen peroxide and molecular oxygen. CAT can detoxify hydrogen peroxide by converting them to oxygen and water. GPx can remove hydrogen peroxide by catalyzing them to water. Our results suggested that CIRBP overexpression significantly suppressed the downregulation of SOD, CAT, and GPx activities induced by A*β*. Thus, CIRBP inhibits oxidative damage-induced mitochondrial dysfunction and cell apoptosis.

## 5. Conclusions

In conclusion, we demonstrate a neuroprotective effect of CIRBP against A*β* induced-cytotoxicity through antioxidative and antiapoptotic pathways in rat primary cortical neurons, which may provide a novel therapeutic strategy for amyloid-based AD prevention or therapy. To the best of our knowledge, this is the first study to evaluate the function of CIRBP against A*β*-induced neurotoxicity.

## Data Availability

The appropriate data used to support the findings of this study are included within the article.

## Supplementary Materials

Supplementary Materials Supplemental Figure 1: The production of A*β*1-42 in the cultured rat cortical neurons after the infection of recombinant adenoviruses at the MOI of 0.1. ND: no detection. ^∗∗^*p* < 0.01 vs. Control. Supplemental Figure 2: MTT results showed no toxicity in the control peptide A*β*35-25, and more toxicity is in the intraneuronal A*β*1-42 than A*β*25-35. ^∗∗^*p* < 0.01 vs. NC, ^##^*p* < 0.01 vs. NC+A*β*25-35.
