# Supplementary material for: CIRBP Ameliorates Neuronal Amyloid Toxicity via Antioxidative and Antiapoptotic Pathways in Primary Cortical Neurons
Source: Oxid Med Cell Longev. 2020 Feb 27;2020:2786139. doi: 10.1155/2020/2786139 (PMC7063194; doi:10.1155/2020/2786139)
Supplement: Supplementary Materials — Supplemental Figure 1: The production of Aβ1-42 in the cultured rat cortical neurons after the infection of recombinant adenoviruses at the MOI of 0.1. ND: no detection. ∗∗p < 0.01 vs. Control. Supplemental Figure 2: MTT results showed no toxicity in the control peptide Aβ35-25, and more toxicity is in the intraneuronal Aβ1-42 than Aβ25-35. ∗∗p < 0.01 vs. NC, ##p < 0.01 vs. NC+Aβ25-35. [file 2786139.f1.pdf]

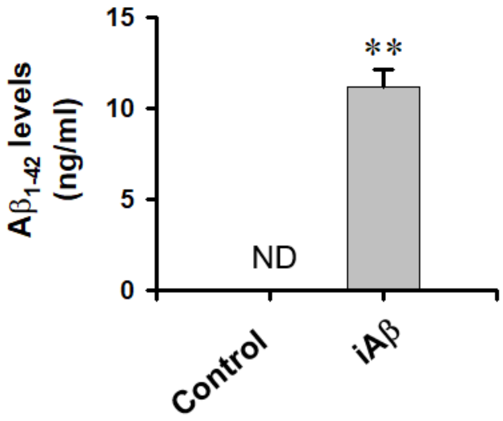

Supplemental Figure 1. The production of Aβ1-42 in the cultured rat cortical neurons after the infection of recombinant adenoviruses at the MOI of 0.1. ND: no detection. **p<0.01 *vs* Control.

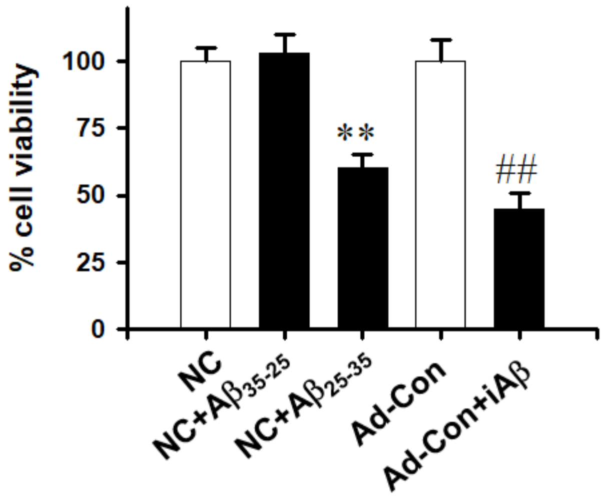

Supplemental Figure 2. MTT results showed no toxicity in the control peptide $A\beta_{35-25}$, and more toxicity is in the intraneuronal $A\beta_{1-42}$ than $A\beta_{25-35}$. **p<0.01 *vs* NC, ## p<0.01 *vs* NC+ $A\beta_{25-35}$.
